# Liraglutide as a treatment option for weight regain after laparoscopic sleeve gastrectomy in patients with obesity and higher anaesthesiologist risk

**DOI:** 10.3389/fsurg.2025.1624455

**Published:** 2025-09-26

**Authors:** Gennaro Martines, Giovanni Tomasicchio, Maria Teresa Rotelli, Michele De Fazio

**Affiliations:** 1 Azienda Ospedaliero Universitaria Policlinico, University of Bari, Bari, Italy; 2 General Surgery Unit, Department of Precision and Regenerative Medicine and Jonic Area (DiMePRe-J), University of Bari Aldo Moro, Bari, Italy; 3 PhD Candidate in Organs and Tissues Transplantation and Cellular Therapies, DiMePRe-J, University of Bari “Aldo Moro”, Bari, Italy; 4 Centro Interdipartimentale di Ricerca Sulle Disfunzioni del Pavimento Pelvico, University of Bari Aldo Moro, Bari, Italy

**Keywords:** anesthesiology risks, GLP-1, liraglutide, obesity, sleeve gastrectomy, weight regain

## Abstract

**Background:**

The underlying causes of weight regain remain largely unclear, and well-defined treatment options are lacking. This retrospective study seeks to assess the effectiveness of liraglutide in managing weight regain among patients with elevated anesthesiology risks who underwent laparoscopic sleeve gastrectomy (LSG) as their primary surgical procedure.

**Methods:**

Clinical records of patients with obesity from January 2017 to January 2024 were retrospectively analysed. Patients with elevated anesthesiology risks who experienced weight regain following LSG and treated with liraglutide 3.0 mg, contingent upon their completion of a follow-up period of six months were included. Demographic data, pre-operative and post-treatment weight, Body Mass Index (BMI), and total weight loss percentage (%TWL) were recorded at the third and sixth months of follow-up.

**Results:**

Forty patients managed weight regain with liraglutide. All patients had an ASA score of 3 and had at least one comorbidity. Pre-treatment weight and BMI were observed at 91 kg and 33.6 kg/m^2^, indicating a marked reduction, with a %TWL of 10.5%. At the 3-month, the median weight decreased to 82 kg, with the BMI at 31 kg/m^2^; *p* < 0.005. At the conclusion of the 6-month, all patients achieved a median weight of 75 kg and a BMI of 28.7 kg/m^2^, demonstrating a significant decline from the 3-month measurements, with a %TWL of 27.5%.

**Conclusion:**

Liraglutide, at a maximum dosage of 3.0 mg, has the potential to mitigate weight regain after LSG in patients who are not appropriate candidates for revisional surgery due to various comorbidities.

## Background

Metabolic surgery is currently recognised as the gold standard treatment for patients with severe obesity, leading to lasting weight loss, enhanced quality of life and remission of obesity-related comorbidities (1, 2). However, up to 20% of bariatric patients experience weight regain or fail to achieve long-term weight loss, which undermines the long-term benefits of the metabolic surgery (3, 4). Despite its growing prevalence, the reasons behind weight regain after bariatric surgery remain unclear, and effective treatment options for this issue are not well established. The literature lacks a clear consensus on how to define weight regain, described as a regain of 10%–25% of excess weight or total weight loss recovery from the lowest weight achieved. This ambiguity complicates the task of accurately estimating the true prevalence of weight regain after bariatric surgery (5–7). The lack of consensus and variability regarding definitions, have resulted in a range of treatment options: lifestyle intervention, endoscopic procedures, revisional surgery, and pharmacological therapy.

Revisional surgery is a valid treatment option for individuals experiencing weight regain. It has become increasingly popular in recent years, now accounting for 16.7% of all bariatric procedures (8). However, such surgeries must be performed in specialized tertiary centres due to their higher complexity and the potential for increased post-operative complications compared to primary surgeries (9). Moreover, in patients with significant anaesthesiology risks, revisional surgery may be contraindicated because studies have shown higher complication rates, both during the perioperative course and in the early post-operative period, compared to primary metabolic surgeries. The mortality rate following revisional surgery is also higher (10, 11).

Pharmacological therapy using GLP-1 analogues may be an effective treatment option for managing weight regain after metabolic surgery (12). Metabolic surgery affects the gastrointestinal signals that regulate satiety and glucose homeostasis, leading to increased circulating levels of the glucagon-like peptide-1 (GLP-1) hormone (13, 14). This hormone has the potential to serve as an early marker for inadequate post-operative weight loss after bariatric surgery. Studies have shown that patients who experience poor weight loss and altered food intake, after bariatric surgery tend to have lower levels of circulating GLP-1 hormone (14).

Liraglutide is a glucagon-like peptide 1 analogue that was approved by the Food and Drug Administration in 2014 for the treatment of obesity. It works by binding to GLP-1 receptors in both the peripheral and central nervous systems, which slows gastric emptying and helps regulate food intake by inducing a feeling of satiety (15, 16). As a standalone therapy, this glucagon-like peptide-1 receptor agonists has shown an effective safety profile, leading to improvements in cardiovascular risk for patients with type 2 diabetes and an average body weight loss of 15%–20% (17, 18). The associated risk profile with this treatment is generally mild and mainly includes gastrointestinal side effects such as nausea, constipation, diarrhea and pancreatitis (19).

Its role in the treatment of weight regain after bariatric surgery remains unclear. Currently, there are only a few studies assessing the efficacy of adjuvant pharmacological treatments for weight regain after bariatric surgery (20–22).

The aim of this retrospective study was to assess the efficacy of liraglutide in addressing weight regain among patients exhibiting elevated anaesthesiology risk who have undergone laparoscopic sleeve gastrectomy (LSG) as their primary metabolic surgical intervention. Within this patient population, revisional surgery may be contraindicated due to an increased risk of complications. Consequently, our study emphasises the potential role of liraglutide in these clinical scenarios.

## Materials and methods

### Study design

A retrospective observational single-centre study was conducted using a prospectively maintained database of patients with obesity who underwent LSG as a primary metabolic surgery at a tertiary referral centre for bariatric treatment. The study took place between April 2017 and March 2024.

### Patient population & eligibility criteria

Patients included in the study were over 18 years of age, had a BMI greater than 40, and presented at least one obesity-related comorbidity, such as hypertension, lipid disorder, OSAS (Obstructive Sleep Apnea Syndrome), cardiovascular disease or diabetes. Additionally, they had an American Society of Anesthesiologists (ASA) score of III. The ASA Physical Status Classification System is a risk stratification tool used to assess a patient's overall health and determine the level of risk associated with surgery. It is a grading system ranging from ASA I (healthy patient) to ASA V (moribund patient). ASA III identified patients with severe systemic disease (23). Those who managed weight regain after LSG treated with liraglutide and completed a minimum follow-up period of 6 months after the pharmacological treatment were also included in the study. Liraglutide was administered to patients with an ASA score of 3 who experienced weight regain following primary metabolic surgery, after undergoing a multidisciplinary evaluation. The exclusion criteria included the following: prior use of a GLP-1 receptor agonist (GLP-1 RA); a family history of medullary thyroid carcinoma; personal or family history of multiple endocrine neoplasia; past history of pancreatitis or acute pancreatitis; alteration of amylase or lipase normal values; liver dysfunction (AST/ALT levels greater than three times the normal value), renal dysfunction (eGFR less than 45 ml/min/1.73 m^2^), ASA scores lower or higher than 3; previous abdominal surgery; acute coronary syndrome within the last 6 months; intolerance or allergy to liraglutide; discontinuation of liraglutide therapy; pregnancy or breastfeeding; and patients with a large hiatal hernia or a hernia larger than 5 cm hernia or a hernia of 5 cm or less associated with severe or intractable gastro-oesophageal reflux symptoms. All patients provided informed consent for surgery following a thorough explanation and counselling regarding the benefits and risks. Consent was also obtained from the patients to participate in the study and to access their electronic medical records for research purposes.

### Outcomes and follow-up

Weight regains was defined *a priori* as a recurrence of ≥10% from the lowest weight achieved after LSG (5). Weight regains was observed and documented during the outpatient follow-up process. This information has been recorded in the SICOB (Società Italiana di Chirurgia dell'Obesità) database for further analysis and reference. The Nadir weight was identified as the lowest weight recorded between 12 and 18 months after primary bariatric surgery (24, 25). Liraglutide was prescribed following a minimum of 12 months after the diagnosis of weight regain. During this period, the patient made efforts to achieve weight loss through a structured diet and regular physical activity, but these attempts were unsuccessful. Liraglutide was administered subcutaneously, using a multidose pen injector, starting with a dose of 0.6 mg per day. The dosage was increased weekly by 0.6 mg until reaching a maximum dose of 3 mg over the course of 24 weeks. Only patients who reached the maximum dose were included in the study. Patients were trained to self-administer the injection. Patients were assessed in an outpatient setting at four weeks following surgery, as well as at three months (with a range of ±10–12 weeks) and at six months (with a range of ±10–24 weeks). All data were recorded in the SICOB database, which is maintained prospectively. Evaluations were conducted by a specialised bariatric nurse under the supervision of a physician, ensuring a comprehensive approach to patient care. Demographic data were collected, including age, gender, preoperative and nadir weight (in kg), pre-operative and nadir BMI (kg/m^2^), and comorbidities. Pre-treatment weight and BMI before Liraglutide therapy, as well as weight and BMI at the 3rd and 6th months of follow-up. Additionally, the nadir percentage of total weight loss (%TWL), and the %TWL before and after treatment were recorded. The %TWL (Percentage of Total Weight Loss) was computed using the following formula: (Initial Weight – Post-treatment Weight)/Initial weight × 100 = % Weight Loss. The primary outcome measured was %TWL, which was assessed at 6 months and 12 months following the treatment.

Approval for conducting this retrospective study was granted by the local hospital Ethics Committee. All investigations were conducted in accordance with the principles of the Declaration of Helsinki.

### Statistical analysis

Continuous parameters were presented as medians with interquartile ranges. Categorical variables were reported as means with percentages. The Comparison of categorical variables was conducted using the Chi-squared test and Fisher's exact test when applicable. Group comparisons were performed using the Wilcoxon test. A *p*-value less than 0.05 was considered statistically significant. Statistical analysis was conducted using RStudio (R version 4.0.3 10/10/2020 Copyright© 2020, The R Foundation for Statistical Computing).

## Results

A total of fifty-two patients with an ASA score of 3, who underwent LSG as primary metabolic surgery, experienced weight regain during follow-up. Four patients were excluded due to prior use of GLP-1 RA, and eight patients were excluded while being treated with liraglutide: three due to intolerance to liraglutide, four for not reaching the maximum dose, and one patient was lost to follow-up. Forty patients (median age 49 years, IQR 41–57, with 90% being women) managed their weight regain with liraglutide and completed a minimum follow-up period of 6 months after starting the pharmacological treatment. All patients had an ASA score of 3 and had at least one comorbidity. The following comorbidities were reported during the initial assessment of patients as follows: OSAS in 32 patients (80%), cardiovascular disease in 27 patients (67.5%), hypertension in 25 (62.5%), type II of diabetes in 21 (52.5%) and lipid disorder in 15 (25%). Baseline characteristics are presented in (Table 1).

**Table 1:** Baseline characteristics of patients.

*n* = 40	
Age (years)	49 (41–57)
Sex	
- M	4 (10%)
- F	36 (90%)
Comorbidity before sleeve	
- Diabetes	21 (52.5%)
- Hypertension	25 (62.5%)
- Lipid disorder	15 (25%)
- OSAS	32 (80%)
- Cardiovascular	27 (67.5%)
- None	0

The median pre-operative weight was 103 kg (IQR 100–110), and the median BMI was 40.5 kg/m^2^ (IQR 40–42). After LSG, the median nadir weight dropped to 71 kg (IQR 70–72), with a median BMI of 27.8 kg/m^2^ (IQR 27–28). This represents a statistically significant decrease in both weight and BMI compared to pre-operative values (*p* < 0.005). The median percentage of total weight loss (%TWL) after surgery was 31.32% (IQR 29.4–33.8%).

**Figure 1:**
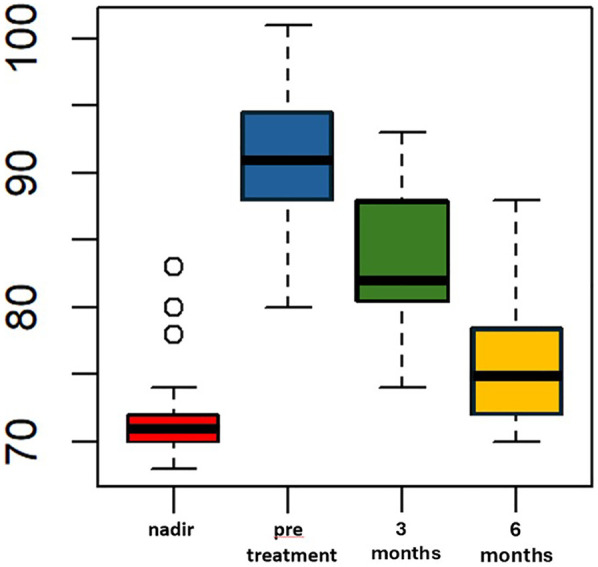
Weight (kg) over time pre and following adjunct treatment with liraglutide 3.0 mg in patients with ASA score of 3. The *x*-axis represents months, while the *y*-axis measures weight in kilograms (kg).

**Figure 2:**
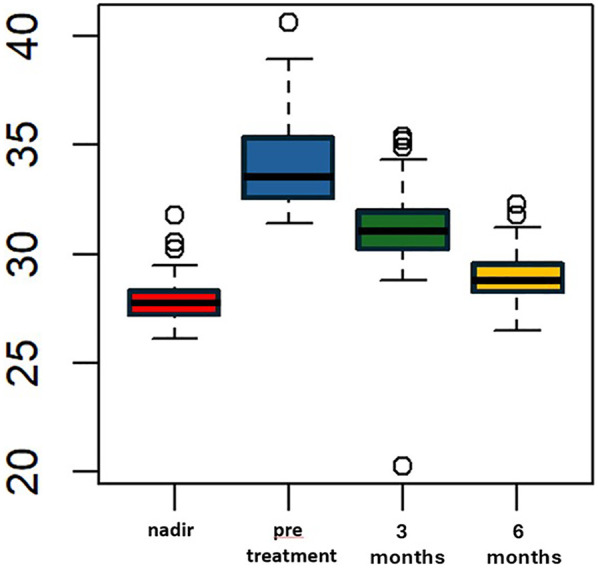
BMI (kg/m^2^) over time pre and following adjunct treatment with liraglutide 3.0 mg in patients with ASA score of 3. The *x*-axis represents months, while the *y*-axis measures weight in kilograms (kg).

Liraglutide treatment was initiated at a median of 69 (IQR 65–73.5) months after LSG, with no adverse events reported. The median pre-treatment weight was 91 kg (IQR 88–94.5) and the median BMI was 33.6 kg/m^2^ (IQR 32.5–35.4). These values were significantly higher than the median nadir weight and BMI (*p* < 0.005). The percentage of total weight loss (%TWL) was 10.5% (IQR 10.1–15.4), which was statistically lower compared to the %TWL at nadir. After 3 months of liraglutide treatment, the median weight decreased to 82 (IQR 80.5–88) kg, which was significantly lower than the median pre-treatment weight (*p* < 0.005). The median BMI at 3 month of treatment was statistically lower than the median pre-treatment BMI, measuring 31 (IQR 30–32) Kg/m^2^ vs. 33.6 (IQR 32.5–35.4) kg/m^2^, *p* < 0.005 (Figure 1). All patients included in the study completed six months of liraglutide treatment. At the end of the treatment period, the median weight was 75 Kg (IQR 72–78.5), and the median BMI was 28.7 (IQR 28–29.6). Both measures were statistically lower compared to the median weight and BMI observed at three months, *p* < 0.005 (Figure 2). Additionally, the median %TWL at six months, at the conclusion of the treatment, was 27.5% (IQR 24.5–30.4). This figure was statistically significantly higher than the median %TWL before treatment (*p* < 0.005).

## Discussion

The recent global increase in the number of patients undergoing bariatric surgery has brought attention to the significant rate of post-operative weight regain after these procedures, highlighting the urgent need for additional treatment options. Currently, the treatment choices for patients experiencing weight regain following primary bariatric surgery are often limited to lifestyle modifications, endoscopic procedures, or revisional surgery. While these surgical techniques have demonstrated some success in addressing weight regain—particularly after LSG—they come with higher complication rates compared to primary surgeries (26–28). The increased risks of marginal ulcers, anastomotic leaks, bleeding, and post-operative mortality may make revisional surgery contraindicated, especially in patients with a high ASA score.

Pharmacological therapy may offer a safer alternative for preventing weight regain in certain patients, withfewer complications and sustained long-term weight reduction (29). Nowadays, no pharmacological weight loss medications are approved for addressing weight regain after primary bariatric surgery, despite interest and evidence supporting medical treatments that could help patients return to their lowest weight, particularly those at higher risk of complications during anaesthesia (30). The first BARI-OPTIMISE trial was the first randomized clinical trial that assessed the efficacy and safety of liraglutide 3.0 mg, compared to placebo, in patients with type 2 diabetes who had experienced poor weight loss at least one year after undergoing Roux-en-Y gastric bypass or LSG. The study reported that over a 24-week period, participants taking liraglutide 3.0 mg experienced a significantly greater reduction in body weight compared to those receiving a placebo (−8.82 vs. −0.54, respectively; *p* < 0.001). The mean difference in percentage body weight change between the liraglutide and placebo groups was– 8.03 (95% CI, −10–39 to −5.66; *p* < 0.01). Additionally, liraglutide treatment was associated with a reduction in body fat mass, favourable changes in cardiometabolic risk factors, and improvements in quality of life.

Furthermore, consistent with our findings, liraglutide was well tolerated, with no severe adverse events, or acute cholecystitis or pancreatitis reported. The only side effects observed were gastrointestinal, including diarrhoea, nausea, constipation and vomiting (31).

Wharton et al., in their retrospective analysis, found that patients who regained weight after various bariatric primary procedures and weretreated with liraglutide 3.0 mg experienced statically significant weight loss, regardless of the type of bariatric surgery they had undergone. Specifically, patients who had gastric sleeve gastrectomy lost an average of 3.6% of their weight, those with gastric banding lost 4.9%, and patients who underwent Roux-en-Y gastric bypass experienced a weight loss of 6.6%. These reductions in weight remained significant even after one year of treatment with Liraglutide, although only the 62.4% of patients reached the recommended maximum clinical dose of 3.0 mg (32). In a recent randomized, double-bind, placebo-controlled trial, Lofton et al. reported that the daily injection of liraglutide was associated with clinically meaningful weight loss in subjects who had regained weight regain after Roux-en-Y bypass. Importantly, there were no severe adverse events reported. In the liraglutide group, 51% of patients achieved a total body weight loss (TBWL) of 10% or more, while 26% reached a TBWL of 15% or more. In contrast, only 3% of patients in the placebo group lost 10% or more of their TBWL, and none achieved a loss of 15% or more (33).

Furthermore, Mohammad et al. investigated the effects of liraglutide specifically in patients who experienced weight regain following sleeve gastrectomy. Their study revealed a statistically significant weight loss of 6.2% after a median liraglutide treatment duration of 3 months, among the 40% of patients who reached the maximum recommended daily dose of 3.0 mg. Patients who tolerated a daily dose of 2.4 mg or higher achieved an greater weight loss of 8.2% (34). In contrast to the other studies in the literature, our cohort consisted exclusively of patients who reached the maximum dose of liraglutide 3.0 mg for 24 weeks to address weight regain. This restricted inclusion criteria may explain our finding of a TWL% of 27.5% after six months of follow-up in patients treated with liraglutide following primary LSG.

Since the approval of Liraglutide by the Food and Drug Administration (FDA) and the European Medicines Agency in 2014 for the treatment of obesity, various novel glucagon-like peptide-1 receptor agonists (GLP-1 RAs) have been introduced, including Semaglutide and Tirzepatide (16, 35).

Semaglutide, which received regulatory approval in 2021, is characterized as a long-acting GLP-1 RA with a half-life of approximately 160 h, thus permitting once-weekly administration. Clinical trials have indicated that a dosage of 2.4 mg of Semaglutide results in a significant weight loss of approximately 12%, in comparison to the 5% weight loss seen with Liraglutide at a dosage of 3.0 mg. Furthermore, Semaglutide has been associated with a substantial reduction in HbA1c levels and a lower incidence of cardiovascular-related mortality. Additionally, it has demonstrated declines in the rates of nonfatal myocardial infarction and nonfatal stroke among patients without diabetes (36, 37).

In 2022, Tirzepatide emerged as the first dual agonist specifically targeting both glucose-dependent insulinotropic peptide (GIP) and the GLP-1 receptor, and it subsequently received FDA approval. This medication is administered via a weekly subcutaneous injection and has demonstrated considerable improvements in weight loss, body mass index (BMI), and waist circumference when compared to prior GLP-1 receptor agonists (38–41). A systematic review conducted by Liu et al. has shown that all dosing regimens of Tirzepatide yielded a statistically significant higher proportion of patients reaching weight loss goals of 5%, 10%, and 15% in comparison to those receiving placebo, insulin, and other GLP-1 receptor agonists. Furthermore, all dosages of Tirzepatide were associated with significant reductions in waist circumference and HbA1c levels. It is important to highlight that higher doses of this innovative dual agonist were linked to an increased risk of overall adverse events, including nausea and diarrhea, relative to traditional GLP-1 receptor agonists. This novel and more effective dual agonist represents a promising option for future studies addressing weight regain in patients with elevated anesthesiological risk (42, 43).

To our knowledge, our retrospective analysis is the first study to report on the management of weight regain in patients with an elevated number of comorbidities and an ASA physical status classification of 3. Our research emphasizes the need to develop innovative strategies that address frailty and disease management in patients with obesity who experience weight regain after primary bariatric surgery. Indeed, the STARSurg Collaborative group, in their prospective, multicentre, international study found that patients undergoing major abdominal surgery with multimorbidity (defined as two or more long-term health conditions) exhibited a higher incidence of frailty compared to those with one or no long-term health condition (3.2% vs. 1.4%; *p* < 0.001). Frailty and ASA physical status levels 3–5 were found to mediate an estimated 31.7% of 30-day mortality in patients with a single long-term health condition. In patients with multimorbidity, this figure increased to an estimated 36.9% (44). Our study emphasizes the importance and safety of GL1-RA in treating weight regain after more than five years from the initial bariatric surgical procedure, particularly in patients with multimorbidity. Furthermore, as demonstrated by our results and various prospective and retrospective trials, no serious adverse events were reported among patients with liraglutide at a dosage of 3.0 mg; only mild gastrointestinal effects were noted.

## Limitations

The main limitations of this study include its single-center design, the limited follow-up duration and its retrospective nature, which may introduce selection bias. Additionally, the absence of a control group and the absence of data for lifestyle change, the small sample size and the focus on only one primary bariatric procedure restrict the ability to draw strong conclusions. Moreover, other GLP-1 receptor agonists, such as semaglutide and tirzepatide, have received approval from the FDA for the treatment of obesity, showing greater weight loss outcomes. However, this study seeks to highlight the efficacy of liraglutide in managing weight regain following LSG in patients identified as having elevated anesthetic risks. Therefore, prospective, multicentre, studies with larger simple sizes and longer follow-up are needed to compare liraglutide with placebo and other GLP1 RA.

## Conclusion

The growing issue of weight regain after primary sleeve gastrectomy has emerged as a significant challenge in bariatric follow-up. This is particularly concerning for patients with multiple health issues and elevated ASA scores, as they face a higher risk of complications following revisional surgery. In the ongoing quest to find effective management strategies for these patients experiencing weight regain, GLP-1 RA may offer a promising solution. Our research indicates that liraglutide at a maximum dosage of 3.0 mg could mitigate weight regain after LSG for patients who are not suitable candidates for revisional surgery due to various comorbidities.

## Data Availability

The raw data supporting the conclusions of this article will be made available by the authors, without undue reservation.
